# Synergistic Effects of Stress-Rupture and Cyclic Loading on Strain Response of Fiber-Reinforced Ceramic-Matrix Composites at Elevated Temperature in Oxidizing Atmosphere

**DOI:** 10.3390/ma10020182

**Published:** 2017-02-15

**Authors:** Longbiao Li

**Affiliations:** College of Civil Aviation, Nanjing University of Aeronautics and Astronautics, No. 29 Yudao St., Nanjing 210016, China; llb451@nuaa.edu.cn; Tel.: +86-25-8489-5963

**Keywords:** ceramic-matrix composites (CMCs), oxidation, matrix multicracking, interface debonding

## Abstract

In this paper, the synergistic effects of stress rupture and cyclic loading on the strain response of fiber-reinforced ceramic-matrix composites (CMCs) at elevated temperature in air have been investigated. The stress-strain relationships considering interface wear and interface oxidation in the interface debonded region under stress rupture and cyclic loading have been developed to establish the relationship between the peak strain, the interface debonded length, the interface oxidation length and the interface slip lengths. The effects of the stress rupture time, stress levels, matrix crack spacing, fiber volume fraction and oxidation temperature on the peak strain and the interface slip lengths have been investigated. The experimental fatigue hysteresis loops, interface slip lengths, peak strain and interface oxidation length of cross-ply SiC/MAS (magnesium alumino-silicate, MAS) composite under cyclic fatigue and stress rupture at 566 and 1093 °C in air have been predicted.

## 1. Introduction

Ceramic materials possess high strength and modulus at elevated temperature. However, their use as structural components is severely limited because of their brittleness. Continuous fiber-reinforced ceramic-matrix composites, by incorporating fibers in ceramic matrices, however, not only exploit their attractive high-temperature strength but also reduce the propensity for catastrophic failure [1]. The critical nature of the application of these advanced materials makes complete characterization necessary. The designers must have information pertaining to not only the strength of the material, but also its fatigue and toughness characteristics. Since ceramic-matrix composites (CMCs) have applications typically in the aerospace industry, these characteristics are especially important due to the severe operating environments encountered and the lower safety factors imposed by the weight considerations. Typically, the tests performed are monotonic loading, cyclic fatigue, and stress rupture at a variety of temperatures and/or atmosphere conditions. Most of the tests are conducted independently. However, these do not accurately represent the loading conditions encountered by an aircraft component. For example, a wing spar will encounter stress rupture–type loading through the flight due to the weight of the airframe it is supporting. It will also encounter cyclic fatigue loading due to the mechanical vibrations from the engines and aerodynamic forces. The temperature will change dramatically with changes in the altitude and flight Mach number. However, the airframe as a whole will not suffer any of these stresses independently, and there will always be some combination of these stresses acting on it at any time. It is possible that the cumulative damage caused to a component under a period of service is some combination of mechanisms caused by the cyclic fatigue, stress rupture and environment (i.e., temperature).

Many researchers have performed experimental and theoretical investigations on the stress rupture and cyclic fatigue behavior of fiber-reinforced CMCs. Lara-Curzio [2] developed a micromechanical model to predict the reliability and lifetime of unidirectional CMCs subjected to stresses beyond the first matrix cracking stress at elevated temperatures. It was shown that the oxidation of the fiber coating leads to changes in the stress distribution of the fibers and exposure of the fibers to the environment, and the oxidation of the fibers leads to fiber strength degradation and ultimately composite fracture. Halverson and Curtin [3] investigated the deformation, strength and stress rupture lifetime of an oxide/oxide CMC at temperatures of 950 °C and 1050 °C without considering oxidative attack, using a stress rupture model incorporating the fiber strength statistics, fiber degradation with time and load, matrix damage, and fiber pullout with the global load-sharing criterion. Sullivan [4] investigated the stress rupture strength of a SiC/SiC composite with a boron nitride (BN) fiber coating in the intermediate temperature range of 700–950 °C. It was found that the stress rupture strength of the composite decreased with increasing time, attributed almost entirely to the slow flaw growth within the fibers. Ruggles-Wrenn et al. [5] investigated the tension-tension fatigue behavior of a two-dimension (2D) woven SiC/SiC composite at 1200 °C in air and in steam environments. The fatigue limit and fatigue lifetime decreased with increasing the loading frequency from 0.1 to 10 Hz in both test environments, and the presence of steam significantly degraded the fatigue performance. Ruggles-Wrenn and Lanser [6] investigated the tension-compression fatigue behavior of a 2D woven Nextel™ 720/alumina composite at 1200 °C in air and in steam. The fatigue limit stress was achieved at 40% and 35% tensile strength in the air and steam environments, respectively, when the maximum cycle number was defined to be 100,000 applied cycles. The presence of steam noticeably degrades the tension-compression fatigue performance of the oxide/oxide composite. Ruggles-Wrenn and Lee [7] investigated the tension-tension fatigue behavior of a 2D woven SiC/SiC composite with an inhibited matrix at 1300 °C in air and in steam conditions. The fatigue limit stress is higher in a steam environment than that in air, which indicates that the presence of steam appears to have a moderately beneficial effect on tension-tension fatigue at 1300 °C. During stress rupture and cyclic loading, the damage evolution inside the composites should be monitored to predict the lifetime. Maillet et al. [8] investigated the damage evolution of a 2D woven SiC/[Si–B–C] composite at temperatures of 450 °C and 500 °C using the acoustic emission (AE)-based approach during static fatigue loading. However, the AE-based approach used for damage monitoring is limited at elevated temperatures. Li [9,10] developed a hysteresis dissipated energy–based damage parameter for the damage evolution and life prediction of fiber-reinforced CMCs under cyclic fatigue loading at room and elevated temperatures.

The objective of this paper is to investigate the synergistic effects of stress rupture and cyclic loading on the strain response of fiber-reinforced CMCs at elevated temperatures in air. The Budiansky–Hutchinson–Evans shear-lag model was used to describe the micro-stress field of the damaged composite considering interface wear and interface oxidation. The statistical matrix multicracking model and fracture mechanics interface debonding criterion were used to determine the matrix crack spacing and interface debonded length. Based on the damage mechanism of interface frictional slipping, the stress-strain relationships were established to analyze the evolution of the peak strain, the interface debonded length and the interface oxidation length. The effects of the stress rupture time, stress level, matrix crack spacing, fiber volume content and oxidation temperature on the peak strain and the interface slip lengths were investigated. The experimental fatigue hysteresis loops, interface slip lengths, peak strain and interface oxidation length of cross-ply SiC/MAS (magnesium alumino-silicate, MAS) composite under cyclic fatigue and stress rupture at 566 and 1093 °C in air were predicted.

## 2. Stress Analysis

**Figure 1:**
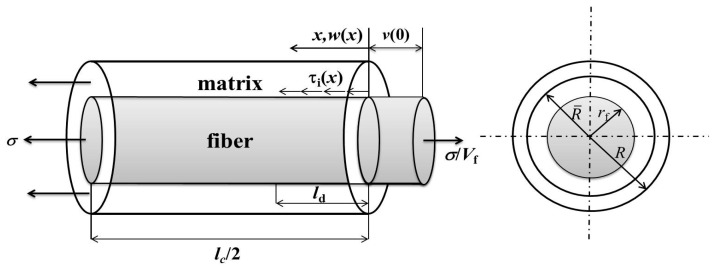
The unit cell of the Budiansky–Hutchinson–Evans shear-lag model.

**Figure 2:**
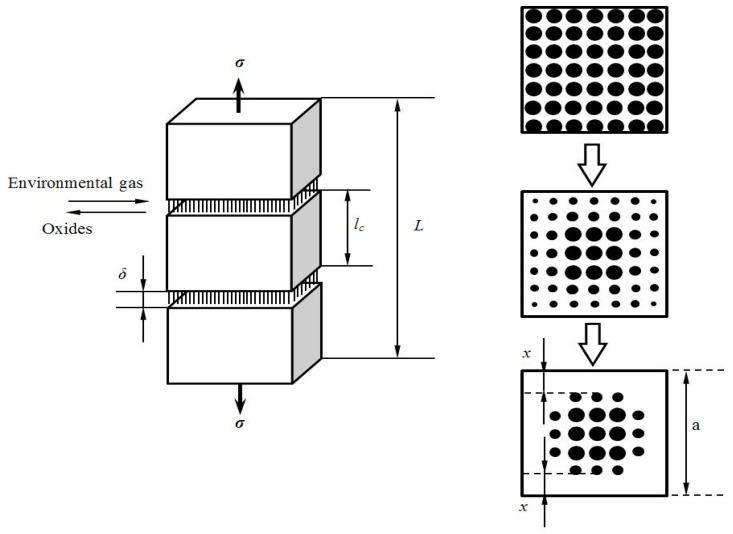
The schematic of fiber oxidation in multiple cracked fiber-reinforced ceramic-matrix composites.

When the applied stress *σ* is higher than the matrix cracking stress, matrix multicracking and interface debonding occur. The unit cell containing a single fiber surrounded by a hollow cylinder of matrix is extracted from the ceramic composite system, as shown in Figure 1. The fiber radius is *r*_f_, and the matrix radius is *R* (*R* = *r*_f_/*V*_f_^1/2^). The length of the unit cell is half the matrix crack spacing *l*_c_/2. Budiansky et al. [11] assumed that the matrix axial load is concentrated at $\bar{R}$, which is an effective radius such that the region between *r*_f_ and $\bar{R}$ only carries the shear stress. At elevated temperatures, matrix cracks will serve as avenues for the ingress of the environment atmosphere into the composite, as shown in Figure 2. The oxygen reacts with the carbon layer along the fiber length at a certain rate of d*ξ*/d*t*, in which *ξ* is the length of the carbon lost in each side of the crack [12].

(1) $$\xi =\varphi_{1}\left[1-\exp\left(-\frac{\varphi_{2}t}{b}\right)\right]$$

where *φ*_1_ and *φ*_2_ are parameters dependent on the temperature and described using the Arrhenius-type laws; *b* is a delay factor considering the deceleration of reduced oxygen activity [12].

Under cyclic loading, the interface shear stress decreases due to interface wear. The interface debonded region can be divided into two regions, including:

1. The interface oxidation region, i.e., *x*∈[0, ξ], where the stress transfer between the fiber and the matrix is controlled by a sliding stress *τ*_i_(*x*) = *τ*_f_.
2. The interface wear region, i.e., *x*∈[*ξ*, *l*_d_], where the stress transfer between the fiber and the matrix is controlled by a sliding stress *τ*_i_(*x*) = *τ*_i_(*N*), in which *τ*_i_(*N*) denotes the interface shear stress at the *N*th applied cycle [13].
  (2) $$\left(\tau_{i}\left(N\right)-\tau_{s}\right)/\left(\tau_{0}-\tau_{s}\right)=\left(1+b_{0}\right){\left(1+b_{0}N^{j}\right)}^{-1}$$
  where *τ*_0_ denotes the initial interface shear stress; *τ*_s_ denotes the steady-state interface shear stress; *b*_0_ is a coefficient; and *j* is an exponent which determines the rate at which interface shear stress drops with the number of cycles *N*.

The axial stress distributions of the fiber, the matrix and the interface shear stress in the interface oxidation region (*x*∈[0, ξ]), the interface wear region (*x*∈[*ξ*, *l*_d_]) and the interface bonded region (*x*∈[*l*_d_, *l*_c_/2]) are given by Equation (3).

(3a) $$\sigma_{f}(x)=\left\{ \begin{array}{l} \frac{\sigma }{V_{f}}-\frac{2\tau_{f}}{r_{f}}x,x\in \left(0,\xi \right)\\ \frac{\sigma }{V_{f}}-\frac{2\tau_{f}}{r_{f}}\xi -\frac{2\tau_{i}\left(N\right)}{r_{f}}\left(x-\xi \right),x\in \left(\xi ,l_{d}\right)\\ \sigma_{\mathrm{fo}}+\left[\frac{V_{m}}{V_{f}}\sigma_{\mathrm{mo}}-2\frac{\tau_{f}}{r_{f}}\xi -2\frac{\tau_{i}\left(N\right)}{r_{f}}\left(l_{d}-\xi \right)\right]\exp\left(-\rho \frac{x-l_{d}}{r_{f}}\right),x\in \left(l_{d},\frac{l_{c}}{2}\right) \end{array}\right.$$

(3b) $$\sigma_{m}(x)=\left\{ \begin{array}{l} 2\frac{V_{f}}{V_{m}}\frac{\tau_{f}}{r_{f}}x,x\in \left(0,\xi \right)\\ 2\frac{V_{f}}{V_{m}}\frac{\tau_{f}}{r_{f}}\xi +2\frac{V_{f}}{V_{m}}\frac{\tau_{i}\left(N\right)}{r_{f}}\left(x-\xi \right),x\in \left(\xi ,l_{d}\right)\\ \sigma_{\mathrm{mo}}-\left[\sigma_{\mathrm{mo}}-2\frac{V_{f}}{V_{m}}\frac{\tau_{f}}{r_{f}}\xi -2\frac{V_{f}}{V_{m}}\frac{\tau_{i}\left(N\right)}{r_{f}}\left(l_{d}-\xi \right)\right]\exp\left(-\rho \frac{x-l_{d}}{r_{f}}\right),x\in \left(l_{d},\frac{l_{c}}{2}\right) \end{array}\right.$$

(3c) $$\tau_{i}(x)=\left\{ \begin{array}{l} \tau_{f},x\in \left(0,\xi \right)\\ \tau_{i}\left(N\right),x\in \left(\xi ,l_{d}\right)\\ \frac{\rho }{2}\left[\frac{V_{m}}{V_{f}}\sigma_{\mathrm{mo}}-2\frac{\tau_{f}}{r_{f}}\xi -\frac{2\tau_{i}\left(N\right)}{r_{f}}\left(l_{d}-\xi \right)\right]\exp\left(-\rho \frac{x-l_{d}}{r_{f}}\right),x\in \left(l_{d},\frac{l_{c}}{2}\right) \end{array}\right.$$

where *V*_m_ denotes the matrix volume fraction; *ρ* denotes the shear-lag model parameter; and *σ*_fo_ and *σ*_mo_ denote the fiber and matrix axial stress in the interface bonded region, respectively.

(4a) $$\sigma_{\mathrm{fo}}=\frac{E_{f}}{E_{c}}\sigma +E_{f}\left(\alpha_{c}-\alpha_{f}\right)\Delta Τ$$

(4b) $$\sigma_{\mathrm{mo}}=\frac{E_{m}}{E_{c}}\sigma +E_{m}\left(\alpha_{c}-\alpha_{m}\right)\Delta Τ$$

where *E*_f_, *E*_m_ and *E*_c_ denote the fiber, matrix and composite elastic modulus, respectively; *α*_f_, *α*_m_ and *α*_c_ denote the fiber, matrix and composite thermal expansion coefficients, respectively; and ∆T denotes the temperature difference between the fabricated temperature T_0_ and room temperature T_1_ (∆T = T_1_ − T_0_).

## 3. Damage Models

### 3.1. Matrix Multicracking

Upon loading of CMCs, cracks typically initiate within the matrix since the strain-to-failure of the matrix is usually less than that of the fiber. With increasing applied stress, the matrix cracking density increases and eventually approaches saturation. The brittle nature of the matrix material and the possible formation of the initial crack distribution throughout the microstructure suggest that a statistical approach to matrix multicracking evolution is warranted in CMCs. The tensile strength of the brittle matrix is assumed to be described by the two-parameter Weibull distribution where the probability of the matrix failure *P*_m_ is determined by Equation (5) [14].

(5) $$P_{m}=1-\exp\left\{-{\left[\frac{\sigma -\left(\sigma_{\mathrm{mc}}-\sigma_{\mathrm{th}}\right)}{\left(\sigma_{R}-\sigma_{\mathrm{th}}\right)-\left(\sigma_{\mathrm{mc}}-\sigma_{\mathrm{th}}\right)}\right]}^{m}\right\}$$

where *σ*_R_ denotes the matrix cracking characteristic strength; *σ*_mc_ denotes the first matrix cracking stress; *σ*_th_ denotes the matrix thermal residual stress; and *m* denotes the matrix Weibull modulus.

**Figure 3:**
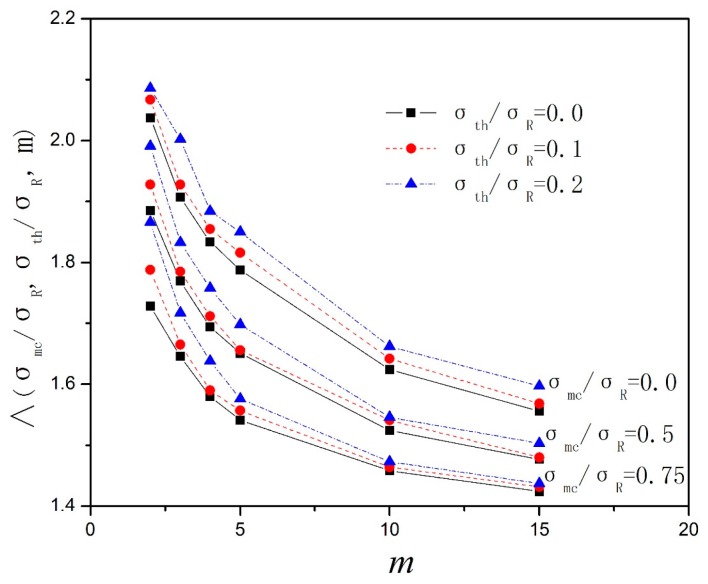
The final nominal matrix crack spacing versus the matrix Weibull modulus of various *σ*_mc_/*σ*_R_ and *σ*_th_/*σ*_R_.

To estimate the instantaneous matrix crack space with the increase of applied stress, it leads to the form of Equation (6).

(6) $$P_{m}=l_{\mathrm{sat}}/l_{c}$$

where

(7) $$l_{\mathrm{sat}}=\Lambda \left(\sigma_{\mathrm{mc}}/\sigma_{R},\sigma_{\mathrm{th}}/\sigma_{R},m\right)\delta_{R}$$

where Λ denotes the final nominal crack space. The final nominal crack space versus the matrix Weibull modulus is simulated by the Monte Carlo simulation method when *σ*_mc_/*σ*_R_ = 0, 0.5, 0.75 and *σ*_th_/*σ*_R_ = 0, 0.1, 0.2 are plotted in Figure 3; *δ*_R_ denotes the characteristic interface sliding length.

(8) $$\delta_{R}=r_{f}\frac{V_{m}E_{m}}{V_{f}E_{c}}\frac{\sigma_{R}}{2\tau_{i}\left(N\right)}$$

Using Equations (5)–(8), the instantaneous matrix crack space is derived by Equation (9).

(9) $$l_{c}=r_{f}\frac{V_{m}E_{m}}{V_{f}E_{c}}\frac{\sigma_{R}}{2\tau_{i}\left(N\right)}\Lambda {\left\{1-\exp\left[-{\left(\frac{\sigma -\left(\sigma_{\mathrm{mc}}-\sigma_{\mathrm{th}}\right)}{\left(\sigma_{R}-\sigma_{\mathrm{th}}\right)-\left(\sigma_{\mathrm{mc}}-\sigma_{\mathrm{th}}\right)}\right)}^{m}\right]\right\}}^{-1}$$

### 3.2. Interface Debonding

When the matrix crack propagates to the fiber/matrix interface, it deflects along the interface. The fracture mechanics approach is adopted in the present analysis. The interface debonding criterion is given by Equation (10) [15].

(10) $$\zeta_{d}=\frac{F}{4\pi r_{f}}\frac{\partial w_{f}\left(0\right)}{\partial l_{d}}-\frac{1}{2}\int_{0}^{l_{d}}\tau_{i}\left(x\right)\frac{\partial v\left(x\right)}{\partial l_{d}}dx$$

where *ζ*_d_ denotes the interface debonded energy; *F*( = *πr*_f_^2^*σ*/*V*_f_) denotes the fiber load at the matrix cracking plane; *w*_f_(0) denotes the fiber axial displacement on the matrix cracking plane; and *v*(*x*) denotes the relative displacement between the fiber and the matrix.

The axial displacements of the fiber and the matrix, i.e., *w*_f_(*x*) and *w*_m_(*x*), are given by Equation (11).

(11a) $$\begin{array}{l} w_{f}\left(x\right)=\int_{x}^{l_{c}/2}\frac{\sigma_{f}\left(x\right)}{E_{f}}dx\\ =\frac{\sigma }{V_{f}E_{f}}\left(l_{d}-x\right)-\frac{\tau_{f}}{r_{f}E_{f}}\left(2\xi l_{d}-\xi^{2}-x^{2}\right)-\frac{\tau_{i}\left(N\right)}{r_{f}E_{f}}{\left(l_{d}-\xi \right)}^{2}+\frac{\sigma_{\mathrm{fo}}}{E_{f}}\left(\frac{l_{c}}{2}-l_{d}\right)\\ +\frac{r_{f}}{\rho E_{f}}\left[\frac{V_{m}}{V_{f}}\sigma_{\mathrm{mo}}-\frac{2\tau_{f}}{r_{f}}\xi -\frac{2\tau_{i}\left(N\right)}{r_{f}}\left(l_{d}-\xi \right)\right]\left[1-\exp\left(-\rho \frac{l_{c}/2-l_{d}}{r_{f}}\right)\right] \end{array}$$

(11b) $$\begin{array}{l} w_{m}\left(x\right)=\int_{x}^{l_{c}/2}\frac{\sigma_{m}\left(x\right)}{E_{m}}dx\\ =\frac{V_{f}\tau_{f}}{r_{f}V_{m}E_{m}}\left(2\xi l_{d}-\xi^{2}-x^{2}\right)+\frac{V_{f}\tau_{i}\left(N\right)}{r_{f}V_{m}E_{m}}{\left(l_{d}-\xi \right)}^{2}+\frac{\sigma_{\mathrm{mo}}}{E_{m}}\left(\frac{l_{c}}{2}-l_{d}\right)\\ -\frac{r_{f}}{\rho E_{m}}\left[\sigma_{\mathrm{mo}}-2\frac{V_{f}\tau_{f}}{r_{f}V_{m}}\xi -2\frac{V_{f}\tau_{i}\left(N\right)}{r_{f}V_{m}}\left(l_{d}-\xi \right)\right]\left[1-\exp\left(-\rho \frac{l_{c}/2-l_{d}}{r_{f}}\right)\right] \end{array}$$

The relative displacement between the fiber and the matrix, i.e., *v*(*x*), is determined by Equation (12).

(12) $$\begin{array}{l} v\left(x\right)=\left|w_{f}\left(x\right)-w_{m}\left(x\right)\right|\\ =\frac{\sigma }{V_{f}E_{f}}\left(l_{d}-x\right)-\frac{E_{c}\tau_{f}}{r_{f}V_{m}E_{m}E_{f}}\left(2\xi l_{d}-\xi^{2}-x^{2}\right)-\frac{E_{c}\tau_{i}\left(N\right)}{r_{f}V_{m}E_{m}E_{f}}{\left(l_{d}-\xi \right)}^{2}\\ +\frac{r_{f}E_{c}}{\rho V_{m}E_{m}E_{f}}\left[\sigma_{\mathrm{mo}}-2\frac{\tau_{f}}{r_{f}}\xi -2\frac{\tau_{i}\left(N\right)}{r_{f}}\left(l_{d}-\xi \right)\right]\left[1-\exp\left(-\rho \frac{l_{c}/2-l_{d}}{r_{f}}\right)\right] \end{array}$$

Substituting *w*_f_(*x* = 0) and *v*(*x*) into Equation (10), it leads to the form of Equation (13).

(13) $$\begin{array}{l} \frac{E_{c}{\left[\tau_{i}\left(N\right)\right]}^{2}}{r_{f}V_{m}E_{m}E_{f}}{\left(l_{d}-\xi \right)}^{2}+\frac{E_{c}{\left[\tau_{i}\left(N\right)\right]}^{2}}{\rho V_{m}E_{m}E_{f}}\left(l_{d}-\xi \right)-\frac{\tau_{i}\left(N\right)\sigma }{V_{f}E_{f}}\left(l_{d}-\xi \right)+\frac{2E_{c}\tau_{f}\tau_{i}\left(N\right)}{r_{f}V_{m}E_{m}E_{f}}\xi \left(l_{d}-\xi \right)\\ -\frac{r_{f}\tau_{i}\left(N\right)\sigma }{2\rho V_{f}E_{f}}+\frac{E_{c}\tau_{f}^{2}}{r_{f}V_{m}E_{m}E_{f}}\xi^{2}+\frac{E_{c}\tau_{f}\tau_{i}\left(N\right)}{\rho V_{m}E_{m}E_{f}}\xi -\frac{\tau_{f}\sigma }{V_{f}E_{f}}\xi +\frac{r_{f}V_{m}E_{m}\sigma^{2}}{4V_{f}^{2}E_{f}E_{c}}-\zeta_{d}=0 \end{array}$$

Solving Equation (13), the interface debonded length *l*_d_ is given by Equation (14).

(14) $$l_{d}=\left(1-\frac{\tau_{f}}{\tau_{i}\left(N\right)}\right)\xi +\frac{r_{f}}{2}\left(\frac{V_{m}E_{m}\sigma }{V_{f}E_{c}\tau_{i}\left(N\right)}-\frac{1}{\rho }\right)-\sqrt{{\left(\frac{r_{f}}{2\rho }\right)}^{2}+\frac{r_{f}V_{m}E_{m}E_{f}}{E_{c}\tau_{i}^{2}}\zeta_{d}}$$

## 4. Stress-Strain Relationship

Under cyclic fatigue loading at elevated temperatures, the interface wear and interface oxidation will affect the degradation of the interface shear stress, the interface debonding and slipping length, and the strain response of fiber-reinforced CMCs. Based on interface debonding and interface slipping between the fiber and the matrix inside of the composite, the interface debonding and slipping can be divided into four different cases, including:

1. Case 1: the interface oxidation region and the interface wear region are less than the matrix crack spacing, and the interface counter-slip upon unloading and the interface new-slip upon reloading are equal to the interface debonded length.
2. Case 2: the interface oxidation region and the interface wear region are less than the matrix crack spacing, and the interface counter-slip upon unloading and the interface new-slip upon reloading are less than the interface debonded length.
3. Case 3: the interface oxidation region and the interface wear region are equal to the matrix crack spacing, and the interface counter-slip upon unloading and the interface new-slip upon reloading are less than the matrix crack spacing.
4. Case 4: the interface oxidation region and the interface wear region are equal to the matrix crack spacing, and the interface counter-slip upon unloading and the interface new-slip upon reloading are equal to the matrix crack spacing.

### 4.1. Case 1

When the interface oxidation region and the interface wear region are less than matrix crack spacing, upon unloading, the interface debonded region can be divided into three regions, i.e., the interface counter-slip region with the interface shear stress of *τ*_f_ (*x*∈[0, ξ]), the interface counter-slip region with the interface shear stress of *τ*_i_(*N*) (*x*∈[*ξ*, *y*]) and the interface slip region with the interface shear stress of *τ*_i_(*N*) (*x*∈[*y*, *l*_d_]), in which *y* denotes the interface counter-slip length. Upon unloading to the unloading transition stress of *σ*_tr_pu_, the interface counter-slip length approaches the interface debonded length, i.e., *y*(*σ* = *σ*_tr_pu_) = *l*_d_. When *σ* < *σ*_tr_pu_, counter-slip occurs over the entire interface debonded region, i.e., *y*(*σ* < *σ*_tr_pu_) = *l*_d_. The unloading strain is divided into two regions, as shown in Equation (15).

(15a) $$\begin{array}{l} \varepsilon_{c\_\mathrm{pu}}=\frac{2\sigma l_{d}}{V_{f}E_{f}l_{c}}+\frac{2\tau_{f}}{r_{f}E_{f}l_{c}}\xi^{2}+\frac{4\tau_{f}}{r_{f}E_{f}l_{c}}\xi \left(l_{d}-\xi \right)+\frac{4\tau_{i}\left(N\right)}{r_{f}E_{f}l_{c}}{\left(y-\xi \right)}^{2}-\frac{2\tau_{i}\left(N\right)}{r_{f}E_{f}l_{c}}{\left(2y-\xi -l_{d}\right)}^{2}\\ +\frac{2\sigma_{\mathrm{fo}}}{E_{f}l_{c}}\left(\frac{l_{c}}{2}-l_{d}\right)+\frac{2r_{f}}{\rho E_{f}l_{c}}\left[\frac{V_{m}}{V_{f}}\sigma_{\mathrm{mo}}+\frac{2\tau_{f}}{r_{f}}\xi +\frac{2\tau_{i}\left(N\right)}{r_{f}}\left(2y-\xi -l_{d}\right)\right]\\ \times \left[1-\exp\left(-\rho \frac{l_{c}/2-l_{d}}{r_{f}}\right)\right]-\left(\alpha_{c}-\alpha_{f}\right)\Delta Τ,\sigma >\sigma_{\mathrm{tr}\_\mathrm{pu}} \end{array}$$

(15b) $$\begin{array}{l} \varepsilon_{c\_\mathrm{pu}}=\frac{2\sigma l_{d}}{V_{f}E_{f}l_{c}}+\frac{2\tau_{f}}{r_{f}E_{f}l_{c}}\xi^{2}+\frac{4\tau_{f}}{r_{f}E_{f}l_{c}}\xi \left(l_{d}-\xi \right)+\frac{4\tau_{i}\left(N\right)}{r_{f}E_{f}l_{c}}{\left(l_{d}-\xi \right)}^{2}-\frac{2\tau_{i}\left(N\right)}{r_{f}E_{f}l_{c}}{\left(l_{d}-\xi \right)}^{2}\\ +\frac{2\sigma_{\mathrm{fo}}}{E_{f}l_{c}}\left(\frac{l_{c}}{2}-l_{d}\right)+\frac{2r_{f}}{\rho E_{f}l_{c}}\left[\frac{V_{m}}{V_{f}}\sigma_{\mathrm{mo}}+\frac{2\tau_{f}}{r_{f}}\xi +\frac{2\tau_{i}\left(N\right)}{r_{f}}\left(l_{d}-\xi \right)\right]\\ \times \left[1-\exp\left(-\rho \frac{l_{c}/2-l_{d}}{r_{f}}\right)\right]-\left(\alpha_{c}-\alpha_{f}\right)\Delta Τ,\sigma <\sigma_{\mathrm{tr}\_\mathrm{pu}} \end{array}$$

where

(16) $$y=\frac{1}{2}\left\{l_{d}+\left(1-\frac{\tau_{f}}{\tau_{i}\left(N\right)}\right)\xi -\left[\frac{r_{f}}{2}\left(\frac{V_{m}E_{m}}{V_{f}E_{c}}\frac{\sigma }{\tau_{i}\left(N\right)}-\frac{1}{\rho }\right)-\sqrt{{\left(\frac{r_{f}}{2\rho }\right)}^{2}+\frac{r_{f}V_{m}E_{m}E_{f}}{E_{c}{\left[\tau_{i}\left(N\right)\right]}^{2}}\zeta_{d}}\right]\right\}$$

Upon reloading, the interface debonded region can be divided into four regions, i.e., the interface new-slip region with the interface shear stress of *τ*_f_ (*x*∈[0, z]), the interface counter-slip region with the interface shear stress of *τ*_f_ (*x*∈[*z*, *ξ*]), the interface counter-slip region with the interface shear stress of *τ*_i_(*N*) (*x*∈[*ξ*, *y*]) and the interface slip region with the interface shear stress of *τ*_i_(*N*) (*x*∈[*y*, *l*_d_]). Upon reloading to the reloading transition stress of *σ*_tr_pr_, the interface new-slip length approaches the interface debonded length, i.e., *z*(*σ*_tr_pr_) = *l*_d_. When *σ* > *σ*_tr_pr_, new-slip occurs over the entire interface debonded length, i.e., *z*(*σ* > *σ*_tr_pr_) = *l*_d_. The reloading strain is divided into two regions, as shown in Equation (17).

(17a) $$\begin{array}{l} \varepsilon_{c\_\mathrm{pr}}=\frac{2\sigma }{V_{f}E_{f}l_{c}}l_{d}-\frac{4\tau_{f}}{r_{f}E_{f}l_{c}}z^{2}+\frac{2\tau_{f}}{r_{f}E_{f}l_{c}}{\left(2z-\xi \right)}^{2}-\frac{4\tau_{f}}{r_{f}E_{f}l_{c}}\left(2z-\xi \right)\left(l_{d}-\xi \right)+\frac{4\tau_{i}\left(N\right)}{r_{f}E_{f}l_{c}}{\left(y-\xi \right)}^{2}\\ -\frac{2\tau_{i}\left(N\right)}{r_{f}E_{f}l_{c}}{\left(2y-\xi -l_{d}\right)}^{2}+\frac{2\sigma_{\mathrm{fo}}}{E_{f}l_{c}}\left(\frac{l_{c}}{2}-l_{d}\right)+\frac{2r_{f}}{\rho E_{f}l_{c}}\left[\frac{V_{m}}{V_{f}}\sigma_{\mathrm{mo}}-\frac{2\tau_{f}}{r_{f}}\left(2z-\xi \right)+\frac{2\tau_{i}\left(N\right)}{r_{f}}\left(2y-\xi -l_{d}\right)\right]\\ \times \left[1-\exp\left(-\rho \frac{l_{c}/2-l_{d}}{r_{f}}\right)\right]-\left(\alpha_{c}-\alpha_{f}\right)\Delta Τ,\sigma <\sigma_{\mathrm{tr}\_\mathrm{pr}} \end{array}$$

(17b) $$\begin{array}{l} \varepsilon_{c\_\mathrm{pr}}=\frac{2\sigma }{V_{f}E_{f}l_{c}}l_{d}-\frac{4\tau_{f}}{r_{f}E_{f}l_{c}}l_{d}^{2}+\frac{2\tau_{f}}{r_{f}E_{f}l_{c}}{\left(2l_{d}-\xi \right)}^{2}-\frac{4\tau_{f}}{r_{f}E_{f}l_{c}}\left(2l_{d}-\xi \right)\left(l_{d}-\xi \right)+\frac{4\tau_{i}\left(N\right)}{r_{f}E_{f}l_{c}}{\left(l_{d}-\xi \right)}^{2}\\ -\frac{2\tau_{i}\left(N\right)}{r_{f}E_{f}l_{c}}{\left(l_{d}-\xi \right)}^{2}+\frac{2\sigma_{\mathrm{fo}}}{E_{f}l_{c}}\left(\frac{l_{c}}{2}-l_{d}\right)+\frac{2r_{f}}{\rho E_{f}l_{c}}\left[\frac{V_{m}}{V_{f}}\sigma_{\mathrm{mo}}-\frac{2\tau_{f}}{r_{f}}\left(2l_{d}-\xi \right)+\frac{2\tau_{i}\left(N\right)}{r_{f}}\left(l_{d}-\xi \right)\right]\\ \times \left[1-\exp\left(-\rho \frac{l_{c}/2-l_{d}}{r_{f}}\right)\right]-\left(\alpha_{c}-\alpha_{f}\right)\Delta Τ,\sigma >\sigma_{\mathrm{tr}\_\mathrm{pr}} \end{array}$$

where

(18) $$z=\frac{\tau_{i}\left(N\right)}{\tau_{f}}\left\{y-\frac{1}{2}\left[l_{d}+\left(1-\frac{\tau_{f}}{\tau_{i}\left(N\right)}\right)\xi -\left[\frac{r_{f}}{2}\left(\frac{V_{m}E_{m}}{V_{f}E_{c}}\frac{\sigma }{\tau_{i}\left(N\right)}-\frac{1}{\rho }\right)-\sqrt{{\left(\frac{r_{f}}{2\rho }\right)}^{2}+\frac{r_{f}V_{m}E_{m}E_{f}}{E_{c}{\left[\tau_{i}\left(N\right)\right]}^{2}}\zeta_{d}}\right]\right]\right\}$$

### 4.2. Case 2

When the interface oxidation region and the interface wear region are less than the matrix crack spacing, upon unloading to the fatigue valley stress, the interface counter-slip length is less than the interface debonded length, i.e., *y*(*σ* = *σ*_min_) < *l*_d_, and the unloading strain is determined by Equation (15a). Upon reloading to the fatigue peak stress, the interface new-slip length is less than the interface debonded length, *z*(*σ* = *σ*_max_) < *l*_d_, and the reloading strain is determined by Equation (17a).

### 4.3. Case 3

When the interface oxidation region and the interface wear region are equal to the matrix crack spacing, upon unloading to the fatigue valley stress, the interface counter-slip length is less than half the matrix crack spacing, i.e., *y*(*σ* = *σ*_min_) < *l*_c_/2. The unloading strain is determined by Equation (19).

(19) $$\varepsilon_{c\_\mathrm{fu}}=\frac{\sigma }{V_{f}E_{f}}-\frac{2\tau_{f}}{r_{f}E_{f}l_{c}}\xi^{2}+\frac{2\tau_{f}}{r_{f}E_{f}}\xi +\frac{4\tau_{i}\left(N\right)}{r_{f}E_{f}l_{c}}{\left(y-\xi \right)}^{2}-\frac{2\tau_{i}\left(N\right)}{r_{f}E_{f}l_{c}}{\left(2y-\xi -\frac{l_{c}}{2}\right)}^{2}-\left(\alpha_{c}-\alpha_{f}\right)\Delta Τ$$

where

(20) $$y=\left[1-\frac{\tau_{f}}{\tau_{i}\left(N\right)}\right]\xi +\frac{r_{f}V_{m}E_{m}}{4V_{f}E_{c}\tau_{i}\left(N\right)}\left(\sigma_{\max}-\sigma \right)$$

Upon reloading to the fatigue peak stress, the interface new-slip length is less than half the matrix crack spacing, i.e., *z*(*σ* = *σ*_max_) < *l*_c_/2. The reloading strain is determined by Equation (21).

(21) $$\begin{array}{l} \varepsilon_{c\_\mathrm{fr}}=\frac{\sigma }{V_{f}E_{f}}-\frac{4\tau_{f}}{r_{f}E_{f}l_{c}}z^{2}+\frac{2\tau_{f}}{r_{f}E_{f}l_{c}}{\left(2z-\xi \right)}^{2}-\frac{4\tau_{f}}{r_{f}E_{f}l_{c}}\left(2z-\xi \right)\left(y-\xi \right)+\frac{4\tau_{i}\left(N\right)}{r_{f}E_{f}l_{c}}{\left(y-\xi \right)}^{2}\\ -\frac{4\tau_{f}}{r_{f}E_{f}l_{c}}\left(2z-\xi \right)\left(\frac{l_{c}}{2}-y\right)-\frac{2\tau_{i}\left(N\right)}{r_{f}E_{f}l_{c}}{\left(2y-\xi -\frac{l_{c}}{2}\right)}^{2}-\left(\alpha_{c}-\alpha_{f}\right)\Delta Τ \end{array}$$

where

(22) $$z=y\left(\sigma_{\min}\right)-\frac{r_{f}V_{m}E_{m}}{4V_{f}E_{c}\tau_{i}\left(N\right)}\left(\sigma_{\max}-\sigma \right)$$

### 4.4. Case 4

When the interface oxidation region and the interface wear region are equal to the matrix crack spacing, upon unloading to the transition stress of *σ*_tr_fu_, the interface counter-slip length approaches half the matrix crack spacing, i.e., *y*(*σ* = *σ*_tr_fu_) = *l*_c_/2. When *σ* > *σ*_tr_fu_, the interface counter-slip length is less than half the matrix crack spacing, i.e., *y*(*σ* > *σ*_tr_fu_) < *l*_c_/2, and the unloading strain is determined by Equation (19). When *σ* < *σ*_tr_fu_, the unloading interface counter-slip occurs over the entire matrix crack spacing, i.e., *y*(*σ* < *σ*_tr_fu_) = *l*_c_/2, and the unloading strain is determined by Equation (23).

(23) $$\varepsilon_{c\_\mathrm{fu}}=\frac{\sigma }{V_{f}E_{f}}-\frac{2\tau_{f}}{r_{f}E_{f}l_{c}}\xi^{2}+\frac{2\tau_{f}}{r_{f}E_{f}}\xi +\frac{4\tau_{i}\left(N\right)}{r_{f}E_{f}l_{c}}{\left(\frac{l_{c}}{2}-\xi \right)}^{2}-\frac{2\tau_{i}\left(N\right)}{r_{f}E_{f}l_{c}}{\left(\frac{l_{c}}{2}-\xi \right)}^{2}-\left(\alpha_{c}-\alpha_{f}\right)\Delta Τ$$

Upon reloading to the transition stress of *σ*_tr_fr_, the interface new-slip length approaches half the matrix crack spacing, i.e., *z*(*σ* = *σ*_tr_fr_) = *l*_c_/2. When *σ* < *σ*_tr_fr_, the interface new-slip length is less than half the matrix crack spacing, i.e., *z*(*σ* < *σ*_tr_fr_) < *l*_c_/2, and the reloading strain is determined by Equation (21). When *σ* > *σ*_tr_fr_, the interface new-slip length occurs over the entire matrix crack spacing, i.e., *z*(*σ*_tr_fr_ < *σ*) = *l*_c_/2, and the reloading strain is determined by Equation (24).

(24) $$\begin{array}{l} \varepsilon_{c\_\mathrm{fr}}=\frac{\sigma }{V_{f}E_{f}}-\frac{4\tau_{f}}{r_{f}E_{f}l_{c}}z^{2}+\frac{2\tau_{f}}{r_{f}E_{f}l_{c}}{\left(l_{c}-\xi \right)}^{2}-\frac{4\tau_{f}}{r_{f}E_{f}l_{c}}\left(l_{c}-\xi \right)\left(\frac{l_{c}}{2}-\xi \right)+\frac{4\tau_{i}\left(N\right)}{r_{f}E_{f}l_{c}}{\left(\frac{l_{c}}{2}-\xi \right)}^{2}\\ -\frac{4\tau_{f}}{r_{f}E_{f}l_{c}}\left(l_{c}-\xi \right)\left(\frac{l_{c}}{2}-y\right)-\frac{2\tau_{i}\left(N\right)}{r_{f}E_{f}l_{c}}{\left(\frac{l_{c}}{2}-\xi \right)}^{2}-\left(\alpha_{c}-\alpha_{f}\right)\Delta Τ \end{array}$$

## 5. Discussions

**Figure 4:**
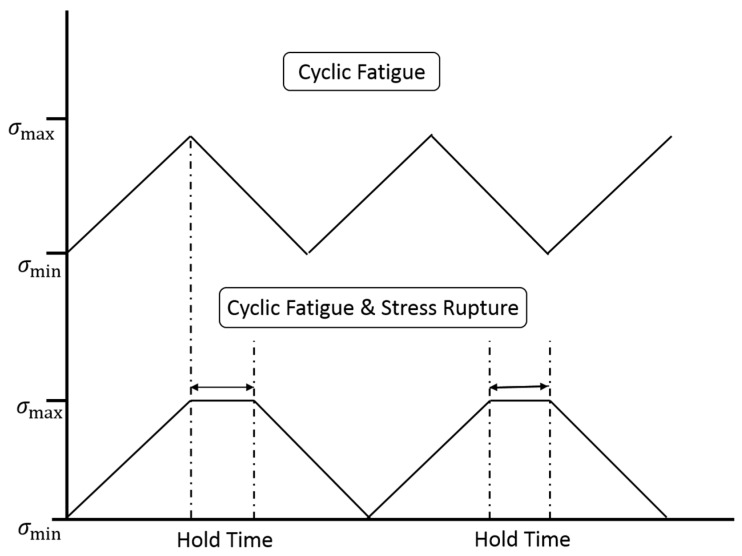
The schematic of cyclic fatigue loading and cyclic fatigue loading with hold time.

Under cyclic loading at elevated temperatures, there are two types of loading sequences considered, as shown in Figure 4, including:

1. Case 1: cyclic fatigue loading without stress rupture, and the interface debonding and frictional slipping between the fiber and the matrix are mainly affected by interface wear.
2. Case 2: cyclic fatigue loading with stress rupture, and the interface debonding and frictional slipping between the fiber and the matrix are mainly affected by interface oxidation.

The synergistic effects of stress rupture and cyclic loading on the strain response of fiber-reinforced CMCs have been investigated, considering different fatigue peak stresses, matrix crack spacings, fiber volume fractions, oxidation temperatures and stress rupture times. The ceramic composite system of unidirectional SiC/MAS [16] was used for the case study and its basic material properties are given by: *V*_f_ = 40%, *r*_f_ = 7.5 μm, *E*_f_ = 200 GPa, *E*_m_ = 138 GPa, *α*_f_ = 4 × 10^−6^/°C, *α*_m_ = 2.4 × 10^−6^/°C, ΔT = −1000 °C, *τ*_i_ = 20 MPa, *τ*_f_ = 5 MPa and *ζ*_d_ = 0.1 J/m^2^.

**Figure 5:**
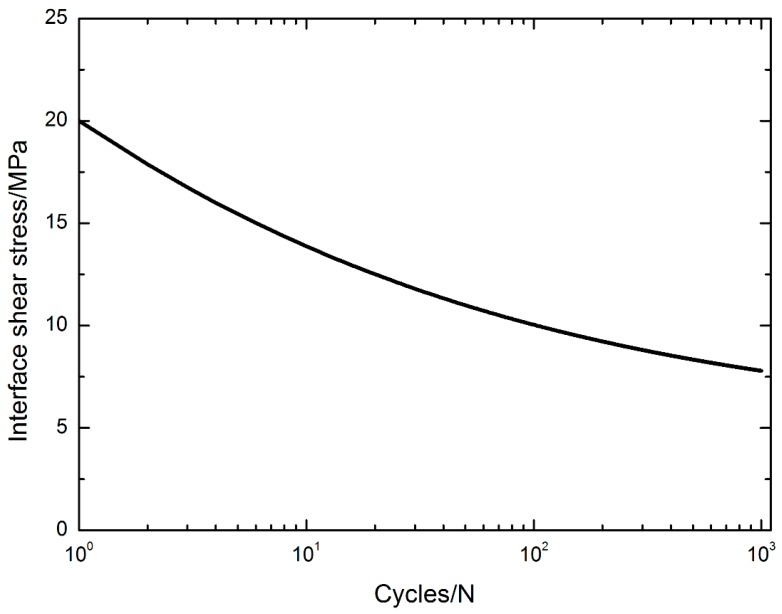
The interface shear stress degradation model.

The interface shear stress versus cycle number curve is illustrated in Figure 5, in which the parameters of the interface shear stress degradation model are given by: *τ*_0_ = 20 MPa, *τ*_s_ = 5 MPa, b_0_ = 4 and j = 0.27. The interface shear stress degrades from 20 MPa at the first applied cycle to 7.8 MPa at the 1000th applied cycle.

### 5.1. Effect of Stress Rupture Time

**Figure 6:**
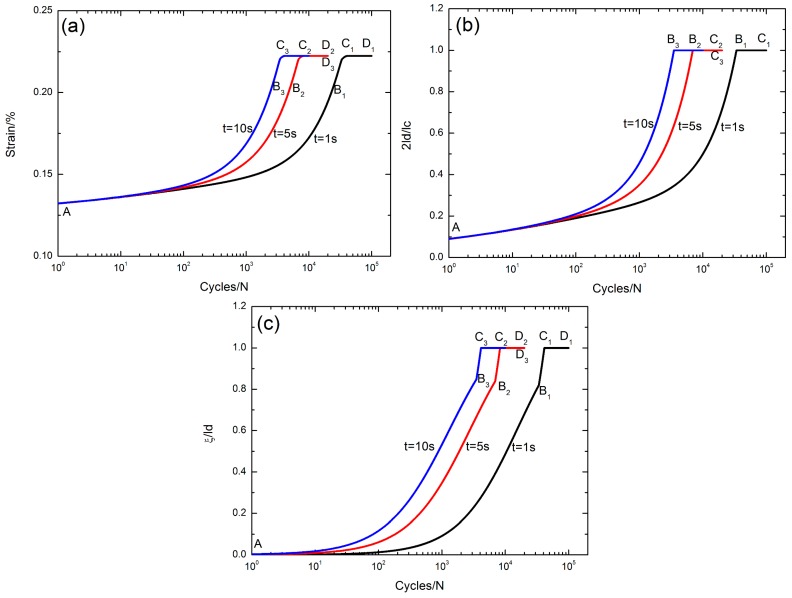
(**a**) The peak strain versus cycle number curves; (**b**) the interface debonded length 2*l*_d_/*l*_c_ versus cycle number curves; and (**c**) the interface oxidation length *ξ*/*l*_d_ versus cycle number curves under *σ*_max_ = 200 MPa corresponding to different stress rupture times t = 1, 5 and 10 s at the oxidation temperature of Tem = 800 °C.

The peak strain *ε*_max_, the interface debonded length 2*l*_d_/*l*_c_, and the interface oxidation length *ξ*/*l*_d_ versus the cycle number curves under *σ*_max_ = 200 MPa, corresponding to different stress rupture times of *t* = 1, 5 and 10 s at the oxidation temperature of Tem = 800 °C, are illustrated in Figure 6.

The evolution of the peak strain versus applied cycles can be divided into three regions, i.e., (1) the peak strain increases with applied cycles, i.e., A–B_1_/A–B_2_/A–B_3_ in Figure 6a, corresponding to the interface partially debonding, i.e., 2*l*_d_/*l*_c_ < 1, A–B_1_/A–B_2_/A–B_3_ in Figure 6b, and the interface partially oxidizing, i.e., *ξ*/*l*_d_ < 1, A–B_1_/A–B_2_/A–B_3_ in Figure 6c when *t* = 1, 5 and 10 s, respectively; (2) the peak strain increases to the peak value, i.e., B_1_–C_1_/B_2_–C_2_/B_3_–C_3_ in Figure 6a, corresponding to the interface completely debonding, i.e., 2*l*_d_/*l*_c_ = 1, B_1_–C_1_/B_2_–C_2_/B_3_–C_3_ in Figure 6b, and the interface partially oxidizing, i.e., *ξ*/*l*_d_ < 1, B_1_–C_1_/B_2_–C_2_/B_3_–C_3_ in Figure 6c when *t* = 1, 5 and 10 s, respectively; and (3) the peak strain remains constant, i.e., C_1_–D_1_/C_2_–D_2_/C_3_–D_3_ part in Figure 6a, corresponding to the interface completely debonding, i.e., 2*l*_d_/*l*_c_ = 1, B_1_–C_1_/B_2_–C_2_/B_3_–C_3_ in Figure 6b, and the interface completely oxidizing, i.e., *ξ*/*l*_d_ = 1, C_1_–D_1_/C_2_–D_2_/C_3_–D_3_ in Figure 6c when *t* = 1, 5 and 10 s, respectively.

With the increasing stress rupture time, the interface slip lengths increase with applied cycles due to interface oxidation, leading to the increased peak strain.

### 5.2. Effect of Stress Levels

**Figure 7:**
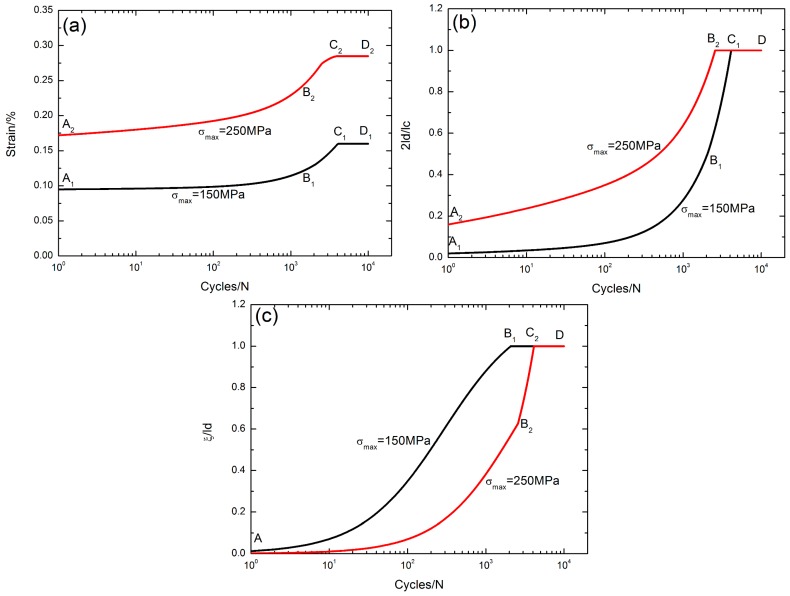
(**a**) The peak strain versus cycle number curves; (**b**) the interface debonded length 2*l*_d_/*l*_c_ versus cycle number curves; and (**c**) the interface oxidation length *ξ*/*l*_d_ versus cycle number curves under fatigue peak stresses of *σ*_max_ = 150 and 250 MPa with stress rupture time t = 10 s at the oxidation temperature of Tem = 800 °C.

The peak strain *ε*_max_, the interface debonded length 2*l*_d_/*l*_c_, and the interface oxidation length *ξ*/*l*_d_ versus the cycle number curves under *σ*_max_ = 150 and 250 MPa with a stress rupture time of *t* = 10 s at the oxidation temperature of Tem = 800 °C are illustrated in Figure 7.

The evolution of the peak strain versus applied cycles can be divided into three regions, i.e., (1) the peak strain increases with applied cycles, i.e., A_1_–B_1_/A_2_–B_2_ in Figure 7a, corresponding to the interface partially debonding, i.e., 2*l*_d_/*l*_c_ < 1, A_1_–B_1_/A_2_–B_2_ in Figure 7b, and the interface partially oxidizing, i.e., *ξ*/*l*_d_ < 1, A–B_1_/A–B_2_ in Figure 7c, when *σ*_max_ = 150 and 250 MPa, respectively; (2) the peak strain increases to the peak value, i.e., B_1_–C_1_/B_2_–C_2_ in Figure 7a, corresponding to the interface partially/completely debonding, i.e., B_1_–C_1_/B_2_–C_1_ in Figure 7b, and the interface completely/partially oxidizing, i.e., B_1_–C_2_/B_2_–C_2_ in Figure 7c, when *σ*_max_ = 150 and 250 MPa, respectively; and (3) the peak strain remains constant, i.e., C_1_–D_1_/C_2_–D_2_ in Figure 7a, corresponding to the interface completely debonding, i.e., 2*l*_d_/*l*_c_ = 1, C_1_–D/B_2_–D in Figure 7b, and the interface completely oxidizing, i.e., *ξ*/*l*_d_ = 1, B_1_–D/C_2_–D in Figure 7c, when *σ*_max_ = 150 and 250 MPa, respectively.

With the increase of the fatigue peak stress, the interface slip lengths increase, leading to the increase of the peak strain.

### 5.3. Effect of Matrix Crack Spacing

**Figure 8:**
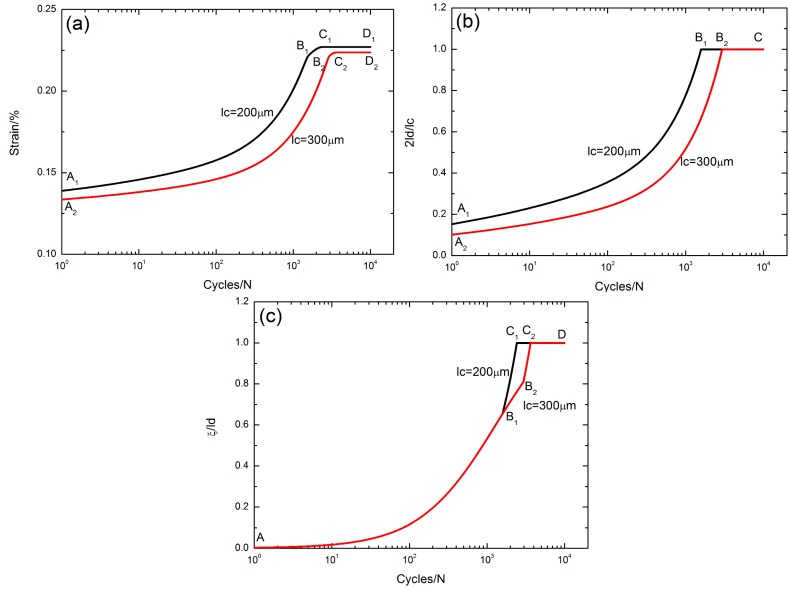
(**a**) The peak strain versus cycle number curves; (**b**) the interface debonded length 2*l*_d_/*l*_c_ versus cycle number curves; and (**c**) the interface oxidation length *ξ*/*l*_d_ versus cycle number curves corresponding to different matrix crack spacings of *l*_c_ = 200 and 300 μm under *σ*_max_ = 200 MPa with stress rupture time of t = 10 s at the oxidation temperature of Tem = 800 °C.

The peak strain *ε*_max_, the interface debonded length 2*l*_d_/*l*_c_, and the interface oxidation length *ξ*/*l*_d_versus the cycle number curves corresponding to different matrix crack spacings of *l*_c_ = 200 and 300 μm under *σ*_max_ = 200 MPa with a stress rupture time of *t* = 10 s at the oxidation temperature of Tem = 800 °C are illustrated in Figure 8.

The evolution of the peak strain versus applied cycles can be divided into three regions, i.e., (1) the peak strain increases with applied cycles, i.e., A_1_–B_1_/A_2_–B_2_ in Figure 8a, corresponding to the interface partially debonding, i.e., 2*l*_d_/*l*_c_ < 1, A_1_–B_1_/A_2_–B_2_ in Figure 8b, and the interface partially oxidizing, i.e., *ξ*/*l*_d_ < 1, A–B_1_/A–B_2_ in Figure 8c, when *l*_c_ = 200 and 300 μm, respectively; (2) the peak strain increases to the peak value, i.e., B_1_–C_1_/B_2_–C_2_ in Figure 8a, corresponding to the interface completely debonding, 2*l*_d_/*l*_c_ = 1, i.e., B_1_–C/B_2_–C in Figure 8b, and the interface partially oxidizing, i.e., B_1_–C_1_/B_2_–C_2_ part in Figure 8c, when *l*_c_ = 200 and 300 μm, respectively; and (3) the peak strain remains constant, i.e., C_1_–D_1_/C_2_–D_2_ in Figure 8a, corresponding to the interface completely debonding, i.e., 2*l*_d_/*l*_c_ = 1, B_1_–C/B_2_–C in Figure 8b, and the interface completely oxidizing, i.e., *ξ*/*l*_d_ = 1, C_1_–D/C_2_–D in Figure 8c, when *l*_c_ = 200 and 300 μm, respectively.

With the increasing matrix crack spacing, the interface slip lengths decrease, leading to the decrease of the peak strain.

### 5.4. Effect of Fiber Volume Content

**Figure 9:**
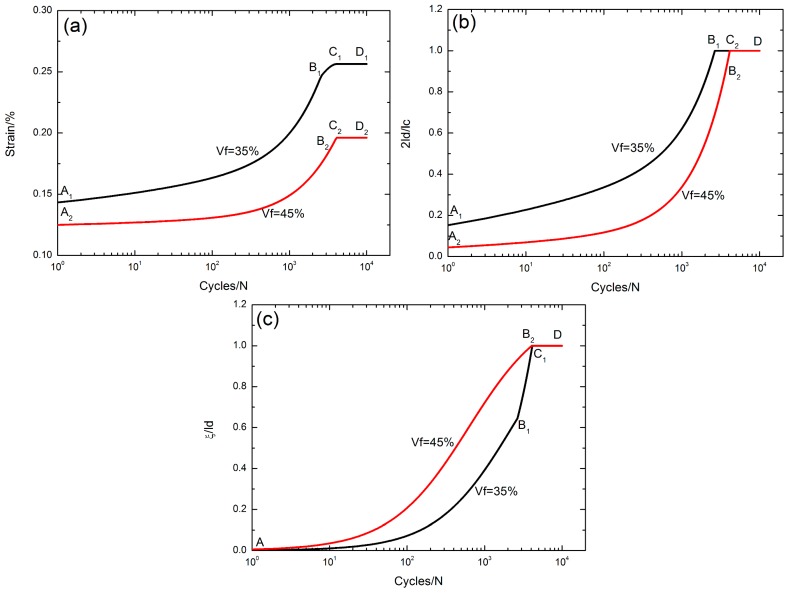
(**a**) The peak strain versus cycle number curves; (**b**) the interface debonded length 2*l*_d_/*l*_c_ versus cycle number curves; and (**c**) the interface oxidation length *ξ*/*l*_d_ versus cycle number curves; corresponding to different fiber volume fractions of *V*_f_ = 35% and 45% under *σ*_max_ = 200 MPa with stress rupture time of 10 s at the oxidation temperature of Tem = 800 °C.

The peak strain *ε*_max_, the interface debonded length 2*l*_d_/*l*_c_, and the interface oxidation length *ξ*/*l*_d_ versus the cycle number curves corresponding to different fiber volume fractions of *V*_f_ = 35% and 45% under *σ*_max_ = 200 MPa with a stress rupture time of *t* = 10 s at the oxidation temperature of Tem = 800 °C are illustrated in Figure 9.

The evolution of the peak strain versus the applied cycles can be divided into three regions, i.e., (1) the peak strain increases with applied cycles, i.e., A_1_–B_1_/A_2_–B_2_ in Figure 9a, corresponding to the interface partially debonding, i.e., 2*l*_d_/*l*_c_ < 1, A_1_–B_1_/A_2_–B_2_ in Figure 9b, and the interface partially oxidizing, i.e., *ξ*/*l*_d_ < 1, A–B_1_/A–B_2_ in Figure 9c, when *V*_f_ = 35% and 45%, respectively; (2) the peak strain increases to the peak value, i.e., B_1_–C_1_/B_2_–C_2_ in Figure 9a, corresponding to the interface completely/partially debonding, i.e., B_1_–C_2_/B_2_–C_2_ in Figure 9b, and the interface partially/completely oxidizing, i.e., B_1_–C_1_/B_2_–D in Figure 9c, when *V*_f_ = 35% and 45%, respectively; and (3) the peak strain remains constant, i.e., C_1_–D_1_/C_2_–D_2_ in Figure 9a, corresponding to the interface completely debonding, i.e., 2*l*_d_/*l*_c_ = 1, B_1_–D/C_2_–D in Figure 9b, and the interface completely oxidizing, i.e., *ξ*/*l*_d_ = 1, C_1_–D/B_2_–D part in Figure 9c, when *V*_f_ = 35% and 45%, respectively.

With the increase of the fiber volume fraction, the interface slip lengths decrease, leading to the decrease of the peak strain.

### 5.5. Effect of Oxidation Temperature

**Figure 10:**
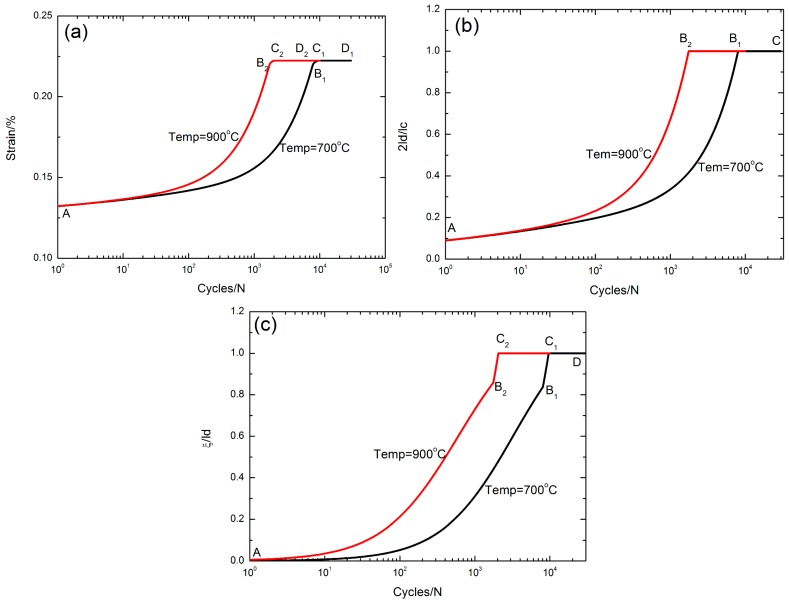
(**a**) The peak strain versus cycle number curves; (**b**) the interface debonded length 2*l*_d_/*l*_c_ versus cycle number curves; and (**c**) the interface oxidation length *ξ*/*l*_d_ versus cycle number curves corresponding to different oxidation temperatures of Tem = 700 °C and 900 °C under *σ*_max_ = 200 MPa with stress rupture time of 10 s.

The peak strain *ε*_max_, the interface debonded length 2*l*_d_/*l*_c_, and the interface oxidation length *ξ*/*l*_d_ versus the cycle number curves corresponding to different oxidation temperatures of Tem = 700 °C and 900 °C under *σ*_max_ = 200 MPa with a stress rupture time of *t* = 10 s are illustrated in Figure 10.

The evolution of the peak strain versus applied cycles can be divided into three regions, i.e., (1) the peak strain increases with applied cycles, i.e., A–B_1_/A–B_2_ in Figure 10a, corresponding to the interface partially debonding, i.e., 2*l*_d_/*l*_c_ < 1, A–B_1_/A–B_2_ in Figure 10b, and the interface partially oxidizing, i.e., *ξ*/*l*_d_ < 1, A–B_1_/A–B_2_ in Figure 10c, when Tem = 700 °C and 900 °C, respectively; (2) the peak strain increases to the peak value, i.e., B_1_–C_1_/B_2_–C_2_ in Figure 10a, corresponding to the interface completely debonding, i.e., B_1_–C/B_2_–C in Figure 10b, and the interface partially oxidizing, i.e., B_1_–C_1_/B_2_–C_2_ in Figure 10c, when Tem = 700 °C and 900 °C, respectively; and (3) the peak strain remains constant, i.e., C_1_–D_1_/C_2_–D_2_ in Figure 10a, corresponding to the interface completely debonding, i.e., 2*l*_d_/*l*_c_ = 1, B_1_–C/B_2_–C in Figure 10b, and the interface completely oxidizing, i.e., *ξ*/*l*_d_ = 1, C_1_–D/C_2_–D in Figure 10c, when Tem = 700 °C and 900 °C, respectively.

With increasing the oxidation temperature, the interface slip lengths increase, leading to the increase of the peak strain.

## 6. Experimental Comparisons

Grant [16] investigated the stress rupture and cyclic fatigue behavior of cross-ply SiC/MAS composite at elevated temperatures in air. The fatigue hysteresis loops, the interface slip lengths, the peak strain and the interface oxidation lengths of cross-ply SiC/MAS composite under different fatigue peak stresses and stress rupture times at elevated temperatures were predicted using the present analysis.

### 6.1. Strain Response under Cyclic Fatigue and Stress Rupture at 566 °C in Air

**Figure 11:**
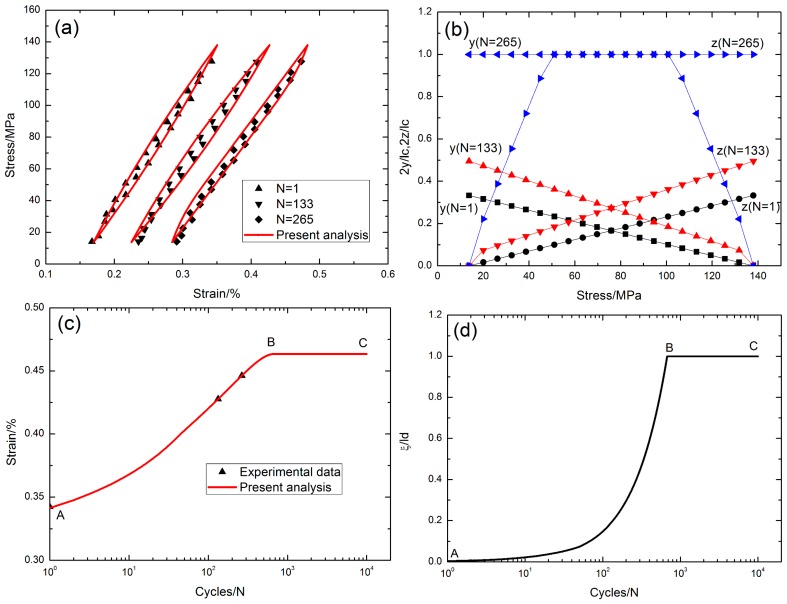
(**a**) The fatigue hysteresis loops corresponding to different applied cycles; (**b**) the interface slip lengths versus applied stress corresponding to different cycle numbers; (**c**) the peak strain versus cycle number curves; and (**d**) the interface oxidation length *ξ*/*l*_d_ versus cycle number curve of cross-ply SiC/MAS composite under *σ*_max_ = 138 MPa at 566 °C with stress rupture time of t = 10 s.

The experimental and theoretical fatigue hysteresis loops under the fatigue peak stress of *σ*_max_ = 138 MPa with the stress rupture time *t* = 10 s are given in Figure 11a. The fatigue hysteresis loops at the cycle numbers of *N* = 1, 133 and 265 corresponded to the interface slip Cases 3, 3 and 4, respectively. The residual strain increased with applied cycles, and the area of fatigue hysteresis loops decreased with the cycle number. The interface slip lengths, i.e., the unloading interface counter-slip length and the reloading interface new-slip length, increased with the cycle number, i.e., from 2*y*/*l*_c_ = 2*z*/*l*_c_ = 0.33 at the first applied cycle to 2*y*/*l*_c_ = 2*z*/*l*_c_ = 1.0 at the 265th applied cycle, as shown in Figure 11b. The experimental and theoretical peak strains versus the cycle number curves are illustrated in Figure 11c. The experimental peak strain increased from 0.34% at the first applied cycle to 0.446% at the 265th applied cycle; the theoretical peak strain increased from 0.34% at the first applied cycle to the peak value of 0.463% at the 677th applied cycle, i.e., the A–B part in Figure 11c, corresponding to the interface completely debonding and the interface partially oxidizing, i.e., the A–B part in Figure 11d. It remained constant at the value 0.463% with increasing applied cycles, i.e., the B–C part in Figure 11c, corresponding to the interface completely debonding and the interface completely oxidizing, i.e., the B–C part in Figure 11d. The theoretical predicted results agreed with the experimental data.

### 6.2. Strain Response under Cyclic Fatigue and Stress-Rupture at 1093 °C in Air

**Figure 12:**
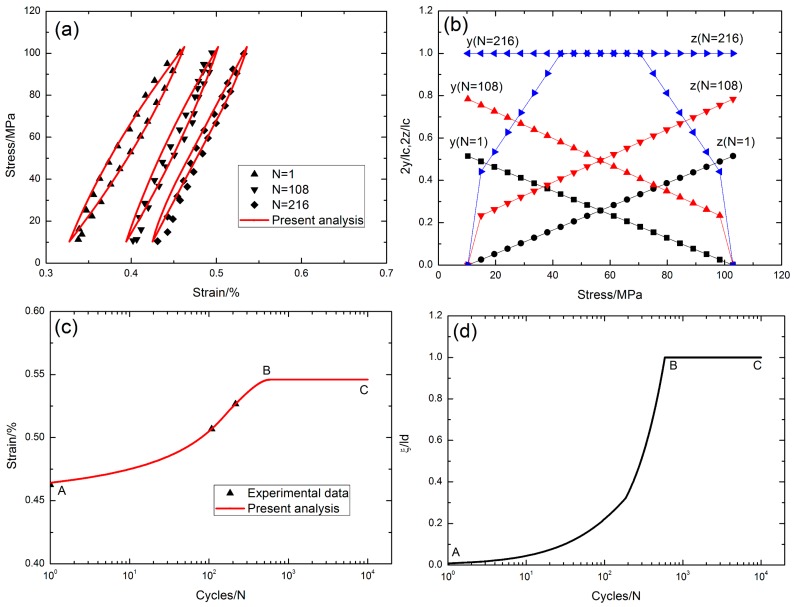
(**a**) The fatigue hysteresis loops corresponding to different applied cycles; (**b**) the interface slip lengths versus applied stress corresponding to different cycle numbers; (**c**) the peak strain versus cycle number curves; and (**d**) the interface oxidation length *ξ*/*l*_d_ versus cycle number curve of cross-ply SiC/MAS composite under *σ*_max_ = 138 MPa at 566 °C with stress rupture time of t = 10 s.

The experimental and theoretical fatigue hysteresis loops under the fatigue peak stress of *σ*_max_ = 103 MPa with the stress rupture time *t* = 10 s are given in Figure 12a. The fatigue hysteresis loops at the cycle numbers of *N* = 1, 108 and 216 corresponded to the interface slip Cased 3, 3 and 4, respectively. The residual strain increased with applied cycles, and the area of fatigue hysteresis loops decreased with the cycle number. The interface slip lengths, i.e., the unloading interface counter-slip length and the reloading interface new-slip length, increased with the cycle number, i.e., from 2*y*/*l*_c_ = 2*z*/*l*_c_ = 0.51 at the first applied cycle to 2*y*/*l*_c_ = 2*z*/*l*_c_ = 1 at the 216th applied cycle, as shown in Figure 12b. The experimental and theoretical peak strains versus cycle number curves are illustrated in Figure 12c. The experimental peak strain increased from 0.46% at the first applied cycle to 0.52% at the 216th applied cycle; the theoretical peak strain increased from 0.46% at the first applied cycle to the peak value of 0.546% at the 590th applied cycle, i.e., the A–B part in Figure 12c, corresponding to the interface completely debonding and the interface partially oxidizing, i.e., the A–B part in Figure 12d. It remained constant at the value of 0.546% with increasing applied cycles, i.e., the B–C part in Figure 12c, corresponding to the interface completely debonding and the interface completely oxidizing, i.e., the B–C part in Figure 12d. The theoretical predicted results agreed with the experimental data.

## 7. Conclusions

The synergistic effects of stress rupture and cyclic loading on the strain response of fiber-reinforced CMCs at elevated temperature were investigated. The effects of the stress rupture time, stress levels, matrix crack spacing, fiber volume fraction and oxidation temperature on the peak strain and the interface slip lengths were investigated. The experimental fatigue hysteresis loops, interface slip lengths, peak strain and interface oxidation length of cross-ply SiC/MAS composite under cyclic fatigue and stress rupture at 566 °C and 1093 °C in air were predicted.

With the increase of the stress rupture time, fatigue peak stress and oxidation temperature, the interface slip lengths increase with applied cycles due to interface oxidation, leading to the increase of the peak strain.

With the increase of the matrix crack spacing and fiber volume fraction, the interface slip lengths decrease, leading to the decrease of the peak strain.
